# Glucose limited feed strategy leads to increased production of fusicocca-2,10(14)-diene by *Saccharomyces cerevisiae*

**DOI:** 10.1186/s13568-018-0662-8

**Published:** 2018-08-22

**Authors:** Lisa Marie Halka, Christian Nowacki, Alica Kleinschmidt, Kevin Koenen, Rolf Wichmann

**Affiliations:** 1 Laboratory of Biochemical Engineering, Department of Biochemical and Chemical Engineering, TU Dortmund University, Emil-Figge-Straße 66, 44227 Dortmund, Germany; 2 Laboratory of Bioprocess Engineering, Department of Biochemical and Chemical Engineering, TU Dortmund University, Emil-Figge-Straße 66, 44227 Dortmund, Germany

**Keywords:** Fusicocca-2,10(14)-diene, Fed-batch fermentation, Feed strategy, Model of feed control, Production improvement, Potential of process development

## Abstract

Fusicocca-2,10(14)-diene (FCdiene) is a diterpene which is interesting as a precursor of the anti-cancer drug fusicoccin A and therefore for pharmaceutical applications. Production of FCdiene using a genetically modified *Saccharomyces cerevisiae* has been previously demonstrated with batch cultivations with a product concentration up to 10 mg/L. However, it is widely known that fed-batch processes can significantly improve product titer in yeast fermentations. This study focuses on the establishment of fed-batch fermentation for FCdiene production because fed-batch cultivations using FeedBeads^®^ indicated that limiting glucose supply could increase yields of biomass (1.07 g_CDW_/g_Glucose_ instead of 0.20 g_CDW_/g_Glucose_) and FCdiene (21.54 mg_FCdiene_/g_Glucose_ instead of 9.74 mg_FCdiene_/g_Glucose_) in shake flask scale and may have implications for larger scale processes. We implemented a new exponential glucose feed profile in a 1.8 L stirred tank reactor. This reduced overfeeding and the consequent, ethanol production. As a result improvements in cell concentrations up to 246% could be achieved and FCdiene yield increased up to 2.8X in the first 28 h. FCdiene concentration reached 161 mg/L and 320 mg/L at 44 h. Fed-batch and batch mode were combined to examine dynamics of bi-modal cultivation where a fed-batch phase was used for biomass production and a batch phase used for FCdiene production potentially supported by ethanol consumption as reported on production of betulinic acid. The present study highlights the potential of process development improvements which increase high-value heterologous diterpene yields from *S. cerevisiae*.

**Electronic supplementary material:**

The online version of this article (10.1186/s13568-018-0662-8) contains supplementary material, which is available to authorized users.

## Introduction

The tricyclic fusicocca-2,10(14)-diene, also known as fusicoccadiene (FCdiene), belongs to the family of diterpenes and is one of the fusicoccanes (Muromtsev et al. 1994). Terpenes are a large group of structurally diverse hydrocarbons derived from the 5-carbon containing building blocks isopentenyl pyrophosphate (IPP) and its isomer dimethylallyl pyrophosphate (DMAPP) (Breitmaier 2005; Ruzicka 1953).

Fusicoccanes have recently gained attention as interesting compounds for their apoptotic and anticancer effects on mammalian cells. Two examples of fusicoccanes with medicinal value are the diterpenoid glucosides fusicoccin A and brassicicene C. Both are synthesized from FCdiene and were first isolated from phytopathogenic fungi (Banerji et al. 1978; Minami et al. 2009). Fusicoccin A was found in *Fusicoccum amygdali* and brassicicene C was isolated from *Alternaria brassicicola*. Both fungi express the same fusicocca-2,10(14)-diene synthase with identical amino acid sequence (Minami et al. 2009; Arens et al. 2013). Due to low natural diterpenoid titers of the fungi, the fusicocca-2,10(14)-diene synthase has been previously expressed in several heterologous microbial hosts, including *Escherichia coli, Aspergillus nidulans* and *Saccharomyces cerevisiae. S. cerevisiae* was found to be the most promising host for FCdiene production (Arens et al. 2013).

Batch fermentations in a 2 L stirred tank reactor have previously shown FCdiene concentrations of approximately 10 mg/L (Arens et al. 2014). This has been optimized in our group up to around 100 mg/L FCdiene while using a 3.1 L stirred tank reactor (Halka and Wichmann 2018). This work presents the investigation of glucose feeding strategies aimed at improving FCdiene yields in fed-batch fermentations. The strategy is a modified protocol based on previously reported processes for amorpha-4.11-diene and artemisinin production (Newman et al. 2006; Tsuruta et al. 2009; Westfall et al. 2012). *S. cerevisiae* growth is known to be more robust in fed-batch mode because of the suppressed Crabtree effect and consequent repression of ethanol production (de Deken 1966). Presented in this work is the development of a model-based glucose feeding strategy which led to a consistent glucose concentration in the fermentation medium which was maintained at concentrations lower than 0.26 g/L throughout the fermentation. In addition, a bi-modal cultivation strategy was investigated which combines fed-batch followed by batch cultivation modes. By starting with a high biomass concentration from the fed-batch phase and ethanol consumption from Crabtree effect, this strategy promoted further increases in FCdiene yield per substrate.

## Materials and methods

The strain used in this work [*S. cerevisiae* CEN.PK2-1C, available at EUROSCARF (30000A) modified from Arens et al. (2014)] is engineered to mimic the one currently used for production of artemisinin (Donald et al. 1997), with a truncated HMG-CoA reductase (tHMGR) as well as the UPC2.1 mutation for increased flux to terpenes.

### Shake flask experiments

For shake flask experiments, 300 mL Erlenmeyer flasks were used with a working volume of 50 mL. The flasks were incubated in a shaking incubator at 30 °C and 120 rpm with shaking amplitude of 2.5 cm. All shake flask experiments lasted 96 h and were performed with four glucose FeedBeads^®^ (Adolf Kühner AG, Basel, Switzerland) per flask. FeedBeads^®^ are slow release discs consisting of polymer particles made from silicon matrix-bound glucose with defined release kinetics, FeedBeads have already been verified for promoting effective and highly reproducible release kinetics in literature. The released glucose amount can be increased proportionally by using more than one glucose FeedBead^®^ (Jeude et al. 2006). The glucose release kinetic of one disc was measured in water (t is the given time in hours).

$$c_{released glucose amount} \left[ {\frac{mg}{disc}} \right] = 1.34 \cdot t^{0.77}$$

For all experiments, the synthetic dropout medium (SD medium) from Arens et al. (2014) was modified. All amino acid concentrations were doubled and 95 mg/L MgCl_2_ (Merck KGaA, Darmstadt, Germany) were added. All components including glucose solutions were sterilized by filtration (PES membrane, 0.2 µm). This medium composition had a pH of ~ 4.0.

### Stirred fermentations

A 3.1 L glass KLF 2000 fermenter (Bioengineering AG, Wald, Switzerland) was used for stirred fermentations with a total working volume of 1.8 L of sterile filtered SD medium. The fermenter was equipped with two six-bladed disk turbine impellers (outer diameter: 48 mm, agitation blade: 15 mm × 12 mm) and four baffles. The temperature was held at 30 °C and the pH was measured in-line. Airflow was regulated at 0.3 L_N_/min using a mass-flow controller (MASS-VIEW^®^, Bronkhorst High-Tech B.V., Ruurlo, The Netherlands). The dissolved oxygen tension (DOT) was measured with a DO-sensor (Bioengineering AG, Wald, Switzerland). Oxygen, carbon dioxide and ethanol concentrations in the exhaust gas were measured online using three gas sensors (BCP-CO_2_, BCP-O_2_ and BCP-EtOH BlueSens gas sensor GmbH, Herten, Germany).

Preparations of precultures were performed in 1 L unbaffled Erlenmeyer flasks with an operating volume of 200 mL and an initial glucose concentration of 0.14 M (25 g/L) for 24 h.

### Batch fermentation

DOT was controlled manually in order to determine the optimal aeration parameters during a fermentation to avoid unintended effects on the measurement of exhaust gas. Initially a stirring rate of 150 rpm was used. The fermentation was inoculated using an initial CDW of 0.12 g/L and 0.14 M (25 g/L) glucose.

### Development of a feed strategy

Using optimal aeration parameters determined in the batch fermentation described above, the airflow was set to 0.3 L_N_/min and the stirring rate was set to 500 rpm to avoid oxygen limitations. An initial glucose concentration of 0.006 M (1 g/L) was used for the first fed-batch fermentation to investigate the glucose consumption during the lag-phase. After complete glucose consumption, glucose was fed in pulses manually. Indicator for a glucose pulse was an increasing DOT signal as well as a decreasing CO_2_ signal in the exhaust gas. A sample was taken and the actual glucose concentration was estimated using a rapid glucose test (Medi-Test Glucose, Macherey–Nagel, Düren, Germany). The volume of the concentrated glucose solution 2.78 M (500 g/L) to be added in order to reach a concentration of maximum 0.1 g/L in the fermentation medium was calculated and added. The fermentation was conducted for 48 h and represented the basis for the calculation of a controlled glucose feed.

For the subsequent fermentation, an exponential feed profile based on the following equation published by Hass and Pörtner (2009) was used.

$$F\left( t \right) = \frac{V(t)}{{\left( {\frac{{c_{S,0} }}{{q_{S} \cdot X(t) \cdot e^{{0.5 \cdot \mu \cdot \Delta t_{F} }} }} - 0.5 \cdot \Delta t_{F} } \right)}}$$

*F*(*t*) describes the glucose feed profile, *V*(*t*) is the working volume, *c*_*S*,0_ is the initial glucose concentration, *q*_*S*_ is the glucose uptake rate which was calculated using previous fermentations (Hass and Pörtner 2009). The CDW is described by *X*(*t*), *μ* is the growth rate and *Δt*_*F*_ is the time interval in which the feed flow rate is constant.

Data of glucose sum curve and biomass were fitted using Microsoft Excel (Microsoft Corporation, Redmond, Washington, USA). However, limitations in the program prevented handling of the measured data, thus Origin^®^ Pro 9.1G (OriginLab Corporation, Northampton, Massachusetts, USA) was applied for satisfactory data representation.

A feed time interval of 30 min was set for the first controlled glucose feed. Initially no glucose was provided; the feed profile was started directly after inoculation. The airflow was set to 0.3 L_N_/min and the initial stirring rate was set to 350 rpm. The growth rate *μ* should be 0.8*μ*_*max*_ as recommended by Hass et al.

### Fed-batch fermentations

The pH was unregulated during experiments to determine the glucose feed strategy. In a second series of experiments pH regulation was implemented for investigation of its influence on growth and FCdiene production. The pH was adjusted to 4.5 with NaOH (0.5 M) or ammonia water (6.65 M). This pH was chosen based on the results of Arens (2015) and our previous studies (Halka and Wichmann 2018). Arens (2015) showed increased FCdiene concentrations and favorable growth of the yeasts in cultivations at pH 4.5–5 using YPD medium. Our results using SD medium showed higher FCdiene levels at lower pH values (Halka and Wichmann 2018). The initial stirring rate was set to 350 rpm. The glucose feed, 2.78 M (500 g/L), was supplied using a Legato™ 210P Syringe Pump (KD Scientific Inc., Holliston, Massachusetts, USA). The fermentations were inoculated using an initial CDW between 0.12 and 0.2 g/L. The initial CDW was adapted to the feed profile because of minimum volume flow rate restriction of the syringe pump.

### Bi-modal fermentation strategy

The fermentations started with a fed-batch phase as above and were subsequently used in batch process after 24 h by stopping the feed pump with a final addition of 5 g/L (0.03 M) glucose. SD medium with a 5X increased yeast nitrogen base concentration was used due to observed limitations in former fermentations. The pH was measured and regulated in the fed-batch phase using ammonia water (6.65 M). Inoculations were conducted with an initial CDW of 0.18 g/L.

### FCdiene quantification

For FCdiene quantification, a gas-chromatographic (GC) system GC-2025 (Shimadzu Corp., Tokyo, Japan) with a Zebron ZB-SemiVolatiles column (length 30 m, inner diameter 0.25 mm, film thickness 0.25 µm) (Phenomenex, Inc., Torrance, California, USA) combined with a flame ionization detector and an auto injector AOC-20i (Shimadzu Corp., Tokyo, Japan) was used. The temperature profile used was: 75 °C for 1 min, increasing to 270 °C at a heating rate of 40 °C/min, and holding at 270 °C for 2 min, followed by an increase to 300 °C at 40 °C/min with a final hold of 3 min. Nitrogen was used as a carrier gas. The system was calibrated using a FCdiene standard kindly provided by the group of Prof. F. Schulz (Ruhr University Bochum, Bochum, Germany) and cycloundecane (Sigma Aldrich, Karlsruhe, Germany) as an internal standard (Arens et al. 2014).

FCdiene was captured from culture broth using adsorption and desorption processes in 3 mL self packed columns filled with adsorbent Europrep 60-60 C18 (0.72 g) for solid phase extraction (SPE) (Europrep 60-60 C18, Dr.-Ing. Herbert Knauer GmbH, Berlin, Germany; empty columns Macherey–Nagel, Düren, Germany). C18 SPE columns showed higher FCdiene recoveries compared to liquid–liquid extractions using *n*-pentane or *n*-heptane. We employed SPE as an extraction protocol due to the scalability of this technology in comparison to solvent based extraction.

Columns were equilibrated using 5 mL aqua bidest., 1 mL ethyl acetate and 5 mL aqua bidest. with a flow rate of 1 mL/min. Columns were dried for 10 min under a vacuum of 0.98 bar using a vacuum manifold (Macherey–Nagel, Düren, Germany) connected to a vacuum pump V-850 (BÜCHI Labortechnik GmbH, Essen, Germany). 2 mL of the cell-free sample was adsorbed six times each through a column at a flow rate of 1 mL/min. Cell-free samples were used because the deficiency of FCdiene bonded to cells is less than the increase in FCdiene concentration from using Europrep 60-60 C18 and to prevent clogging of the adsorbent particles.

After adsorption the columns were washed of bound hydrophilic compounds with 2 mL aqua bidest. FCdiene was eluted using 1 mL ethyl acetate at a flow rate of 1 mL/min. 10 µL of the internal standard cycloundecane (1 g/L) was mixed with 90 µL of the eluate for GC-FID analysis. The FCdiene concentration was calculated based on peak area ratios of FCdiene and internal standard peaks and compared with a calibrated standard curve of FCdiene. An exemplary chromatogram is shown in Additional file 1: Figure S1. Further information for analysis of cell dry weight, pH, glucose, and ethanol concentrations are given in Additional file 1: Materials and methods.

### Yields of fermentations

To compare the fermentations, the substrate specific biomass yield *Y*_*X*/*S*_ and the substrate specific product (FCdiene) yield *Y*_*P*/*S*_ were calculated based on the following two equations.

$$Y_{X/S} = \frac{{c_{X} - c_{X,0} }}{{c_{S,0} - c_{S} }}$$

$$Y_{P/S} = \frac{{c_{P} - c_{P,0} }}{{c_{S,0} - c_{S} }}$$

*c*_*X*_ describes the biomass concentration at the end of the fermentation, *c*_*X*,0_ is the initial biomass concentration, *c*_*P*,0_ is the initial FCdiene concentration and *c*_*P*_ is the final FCdiene concentration. *c*_*S*,0_ is the initial glucose concentration and *c*_*S*_ is the glucose concentration at the end of the fermentation. The difference *c*_*S*,0_ − *c*_*S*_ is calculated from the consumed glucose mass and the reactor volume for fed-batch fermentations.

## Results

### Shake flask experiments

**Fig. 1:**
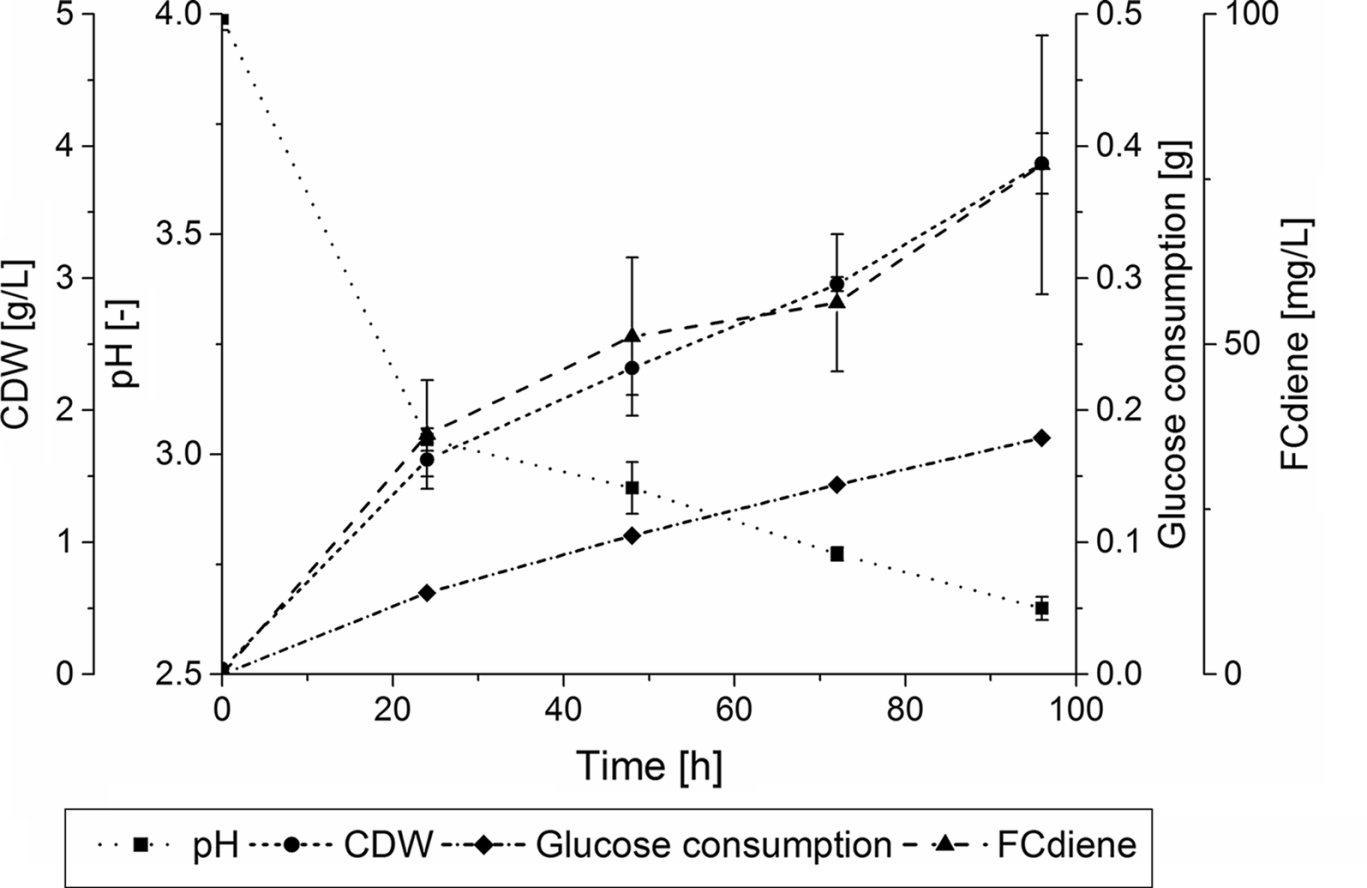
Fed-batch reference fermentation of *S. cerevisiae* growing in SD medium with glucose FeedBeads^®^ as sole carbon source. Cell dry weight concentrations, pH, glucose consumption and FCdiene concentrations are shown. Error bars give the standard deviation of three biological replicates

A typical fermentation strategy for yeasts is the fed-batch fermentation which benefits from reducing the Crabtree effect. This also means that the glucose used should be available for the production of biomass and FCdiene. Therefore, fed-batch shake flask experiments with FeedBeads^®^ were performed as a proof of principle. Using glucose FeedBeads^®^, the biomass concentration reached the same level as in batch fermentations and 77 mg/L FCdiene was reached by 96 h (Fig. 1). The fermentations were performed in triplicate. Therefore, it is possible to synthesize one-third of FCdiene produced in batch fermentation with approximately one-fifth of the glucose (4.1 g/L). Thus the fed-batch process exhibited potential for FCdiene production with decreased substrate consumption and thus indicates higher substrate specific biomass (1.07 g_CDW_/g_Glucose_) and FCdiene (21.54 mg_FCdiene_/g_Glucose_) yields. It indicates a great potential for further fermentations in larger scales.

### Investigation of fermentation regulation and feed strategies

**Fig. 2:**
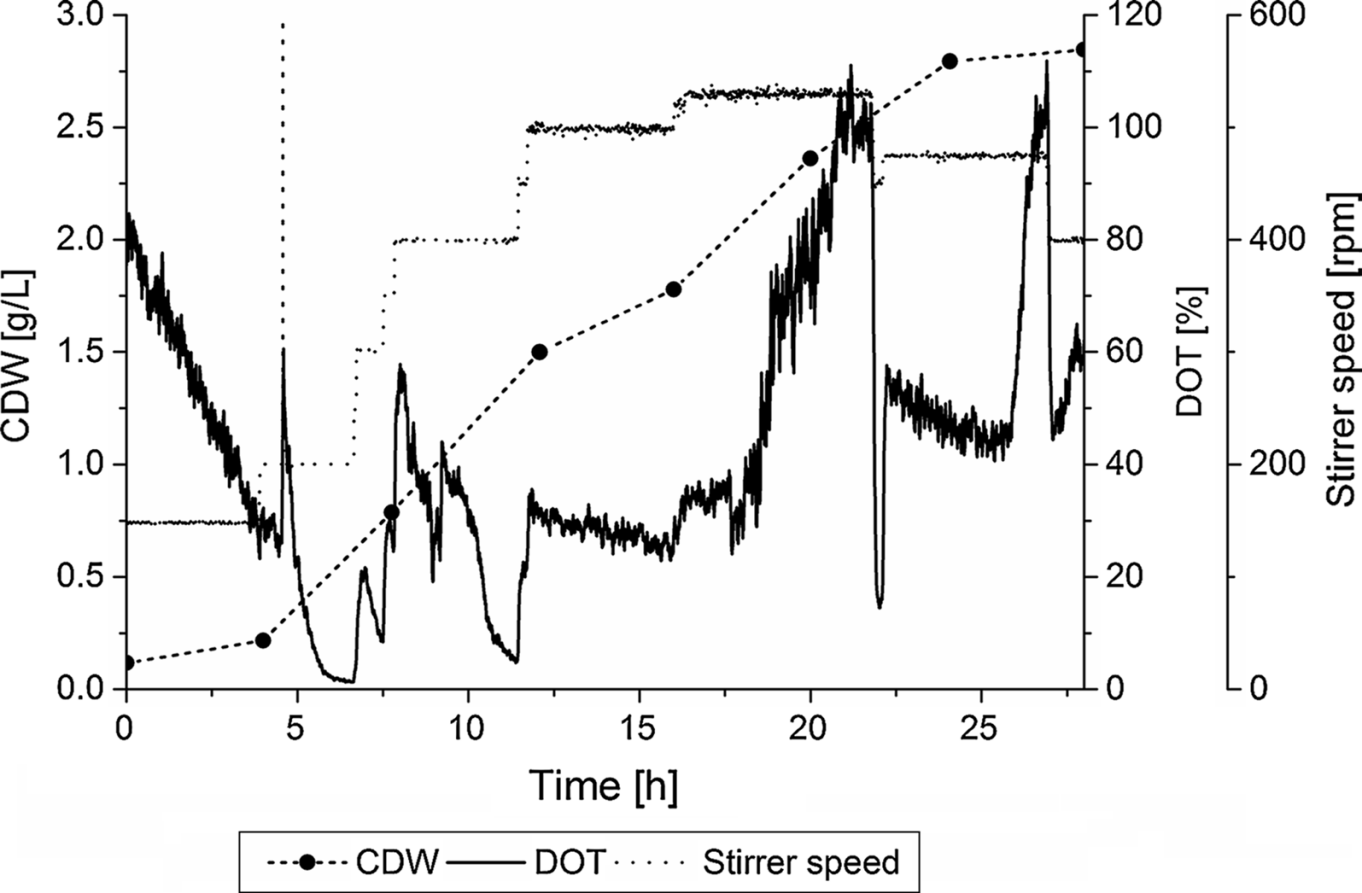
Batch fermentation of *S. cerevisiae* in a KLF2000 fermenter (working volume 1.8 L), using glucose as a carbon source with an unregulated pH in SD medium. Stirring rate was controlled manually to find constant aeration parameter without limitations and without influences on the exhaust gas. Cell dry weight concentrations, DOT and stirring rate are shown

In this study, the influence of fed-batch mode on FCdiene production was investigated. For the development of an optimized fed-batch fermentation strategy iterative changes were made towards improving the process. The first step was investigation of batch fermentation parameters. The aim of these analyses was to minimize the overfeeding of glucose in fed-batch processes by encouraging the use the glucose for growth and mainly for FCdiene production. The batch fermentation was operated for 28 h in order to find a suitable constant stirrer speed which did not influence the exhaust gas and DOT signals (Fig. 2). Figure 2 depicts that oxygen limitation was avoided using a stirrer speed of 500 rpm. Within 24 h, the glucose was completely metabolized and 3 g/L ethanol was produced with FCdiene concentration reaching 52 mg/L. This was more than twofold higher FCdiene concentrations in comparison with previously reports (Arens et al. 2014).

This batch fermentation was followed by manually operated fed-batch fermentation with an initial glucose concentration of 1 g/L (0.006 M). The changes in the CO_2_ and DOT signals were used as an indicator for adding new glucose solution (500 g/L, 2.78 M) up to a glucose concentration of 0.1 g/L in the fermentation medium. With this method, a biomass concentration of 5.4 g/L was reached after 39 h and the glucose concentration was not higher than 0.14 g/L after consumption of the initial 1 g/L within 12 h. Ethanol concentrations up to 1.6 g/L were measured and FCdiene concentrations of 323 mg/L were observed after 44 h. The FCdiene concentration was more than sixfold higher than in the described batch fermentation because the substrate specific product yield was increased 2.8-fold (Table 1) as well. In addition, the final biomass increased nearly twofold and resulted in 16-fold higher FCdiene yields than previously reported (Arens et al. 2014).

**Table 1:** Impact of development of a glucose feed strategy on growth of *S. cerevisiae* and FCdiene production

Fermentations	Name	F1	F2	F3	F4	F5	F6	First fed-batch fermentation
	Description	Batch fermentation	Fed-batch fermentation with manual feed	Fed-batch fermentation with controlled feed and variable stirrer speed	Fed-batch fermentation with controlled feed and staircase-shaped stirrer profile	Fed-batch fermentation with controlled feed and linear stirrer profile	Optimized fed-batch fermentation	
Reference		This study						Arens et al. (2014)
Initial glucose concentration (g/L)		30	1	0	0	0	0	20
Total amount of glucose (g)		52.42	21.53	44.22	74.82	62.26	45.82	nd
*Type of feed*		*None*	*Pulses based on DOT*	*Continuous controlled feed*	*Continuous controlled feed*	*Continuous controlled feed*	*Continuous controlled feed*	*Once 15* g/L
Time interval of const. feedrate (min)		–	–	30	15	15	15	–
*Preset µ*		*–*	*–*	*0.8*_*max*_	*Variable for each interval*	*Variable for each interval*	*Variable for each interval*	*–*
Fermentation time (h)		28	48	48	50	40	48	120
pH		Unregulated	Unregulated	Unregulated	Unregulated	Unregulated	Unregulated	20 mM succinate
Final biomass (g/L)		2.84	5.00	5.50	8.68	8.73	8.34	3.3
*Final FCdiene* (mg/L)		*51.71*	*256.42*	*201.73*	*405.80*	*249.21*	*250.46*	*~ 20*
*Max. glucose* (g/L)		*30.75*	*0.96*	*3.02*	*1.99*	*0.94*	*0.26*	*20*
Max. ethanol (g/L)		9.67	1.64	6.38	7.15	6.25	3.00	nd
Y_X/S_ (g_CDW_/g_Glc_)^a^		0.09	0.36	0.22	0.23	0.25	0.32	nd
q_Glc,max_ (g_Glc_/(g_CDW_ h))		2.50	0.65	0.57	0.50	0.32	0.31	nd
µ_max_ (1/h)		0.30	0.22	0.27	0.27	0.20	0.15	nd
Y_P/S_ (mg_FCdiene_/g_Glc_)^a^		1.78	18.82	8.44	10.74	7.34	9.77	nd
Y_P/X_ (mg_FCdiene_/g_CDW_)^a^		18.95	52.79	37.56	45.94	29.13	30.77	nd
r_FCdiene,X_ (mg_FCdiene_/(g_CDW_ h))^b^		1.27	4.02	1.25	1.43	2.79	1.26	nd
STY (mg_FCdiene_/(L h))		1.85	5.34	4.20	9.92	6.23	5.22	nd

*nd* not determined

Italic values indicates the main changes in the fermentations

^a^Yields and STY were determined at the end of the fermentations

^b^Product formation rates were determined during exponential growth phase

The glucose sum curve and the growth curve of the manually operated fed-batch fermentation were used for the fit and calculation of the feed profile F(t), which was transferred to the syringe pump for the following fed-batch fermentation.

For the first calculated feed, a time interval of 30 min for each flow rate and *μ* = 0.8*μ*_*max*_ were chosen. The target was not to exceed a glucose concentration of 0.1 g/L in the whole fermentation for suppression of the Crabtree effect (de Deken 1966). No glucose was added initially in order to prevent overfeeding in the lag phase. Nevertheless, the overfeeding occurred and a glucose concentration of 0.2 g/L was detected. As the author described, the suggested µ could result in an overfeeding if the actual µ is less than 0.8*μ*_*max*_ (Hass and Pörtner 2009). At the end of the exponential phase, the glucose concentration was found to be 3 g/L, indicating overfeeding throughout the process. The maximum biomass concentration measured in this fermentation was 6.9 g/L and a maximum FCdiene concentration of 217 mg/L was observed. This indicated that the time interval of 30 min is too long for the desired feed profile and the growth rate used for the calculation additionally could be too high.

The procedure of calculating the feed profile was repeated with a time interval of 15 min for further optimization. The constant stirrer profile was changed to a staircase-shaped pattern as an adaptation to the oxygen used by the system. A doubled concentration of yeast nitrogen base was used in order to avoid limitations. In the next fermentation, the constant growth rate µ was modified to a variable growth rate in order to implement different growth phases and reduce overfeeding of glucose. These variable growth rates could be calculated from the fitted glucose sum curve and biomass of the previous fermentation for each feed time interval.

In the late stage of the exponential, as well as the stationary phase, glucose concentration could be decreased to 0.64 g/L on the average. However, glucose was observed to reach 2 g/L at time point of 46 h in the stationary phase. Up to 561 mg/L FCdiene was detected after 47 h and the concentration decreased to under 500 mg/L within the next 2 h (data not shown). The biomass concentration could be increased up to 9.0 g/L, although a limitation of yeast nitrogen base could be observed. This led us to adapt the yeast nitrogen base up to fivefold the concentration described for SD medium.

In the next fed-batch fermentation, a linear stirrer profile was developed based on the staircase-shaped profile. In this fermentation 0.7 g/L glucose concentration was attained and a biomass concentration of 8.5 g/L was reached while 287 mg/L FCdiene was produced.

Due to persistent overfeeding, the glucose consumption curve instead of glucose sum curve was fitted as a last step in the last fermentation. This resulted in maximum measured glucose concentrations of 0.26 g/L and up to 3 g/L ethanol produced from yeast culture. The biomass concentration reached from 8 to 9.1 g/L in the stationary phase and produced up to 284 mg/L FCdiene.

**Fig. 3:**
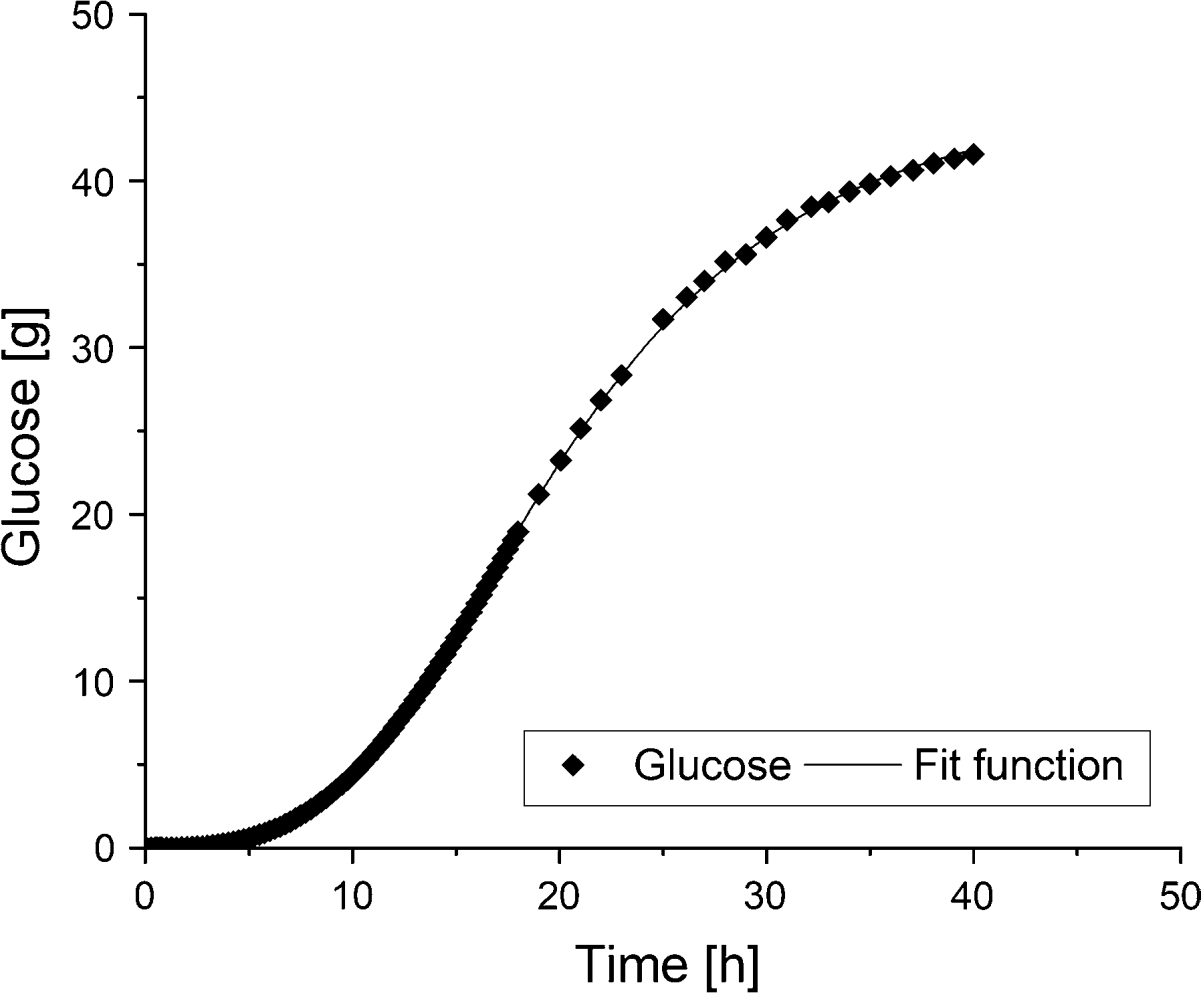
An exemplary fit of the glucose feed of a fed-batch fermentation of *S. cerevisiae* in a KLF2000 fermenter (working volume 1.8 L). Glucose sum curve and mathematical fit with Origin^®^ Pro 9.1 G are shown

An exemplary fit of the glucose feed is shown in Fig. 3. The fit is described by the following equation with an R^2^ of 0.99987.

$$F(t) = 45.9 + \frac{{\left( {0.129 - 45.9} \right)}}{{1 + \left( {\frac{t}{20.0}} \right)^{3.36} }}$$

**Fig. 4:**
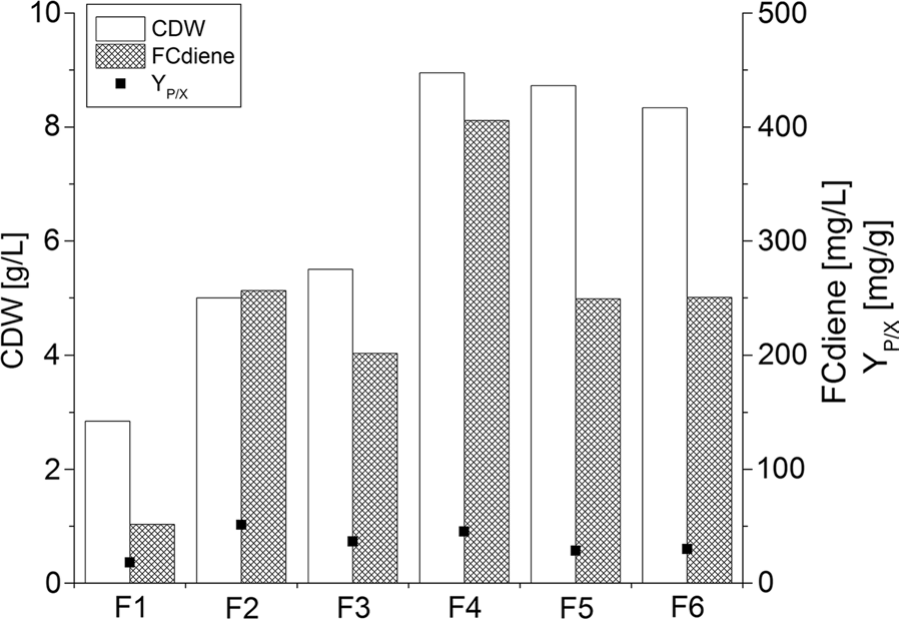
Comparison of batch and fed-batch fermentations: Fermentations in a KLF2000 fermenter (working volume 1.8 L), using glucose as a carbon source with unregulated pH. Cell growth and FCdiene concentrations with calculated cell specific product yield are shown. F1: Batch fermentation, F2: Fed-batch fermentation with manual feed, F3: Fed-batch fermentation with controlled feed and variable stirrer speed, F4: Fed-batch fermentation with controlled feed and staircase-shaped stirrer profile, F5: Fed-batch fermentation with controlled feed and linear stirrer profile, F6: Optimized fed-batch fermentation

The biomass and FCdiene concentrations at the end of each fermentation are shown in Fig. 4. The FCdiene concentration did not increase according to biomass concentration. No direct link between those two parameters could be found, as expected (Table 1). The development indicated that an elongation of the exponential growth phase was not possible although glucose overfeeding could be avoided. Production of around 280 mg/L FCdiene could be achieved.

**Fig. 5:**
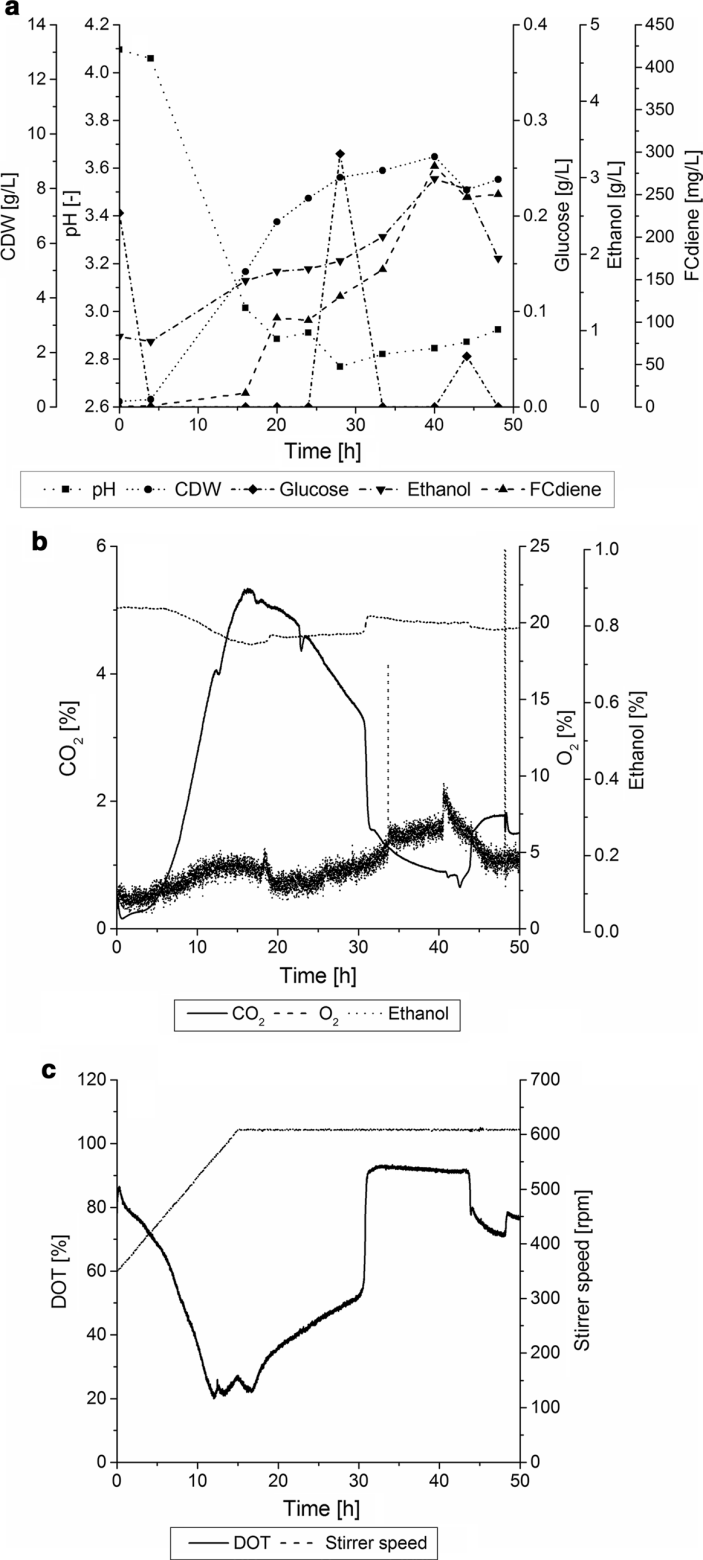
Optimized fed-batch fermentation of *S. cerevisiae* in the KLF2000 fermenter (working volume 1.8 L) using glucose as sole carbon source with unregulated pH. **a** Cell dry weight concentrations, pH, glucose, ethanol and FCdiene concentrations are shown. **b** CO_2_–, O_2_– and ethanol concentrations in exhaust gas are shown. **c** Stirring rate and DOT are shown

The results of the optimized fed-batch fermentation are shown in Fig. 5. A linear stirring profile was developed using an iterative adaptation of a staircase-shaped stirring profile in order to prevent oxygen limitations and prevent shifts in exhaust gas signals.

**Fig. 6:**
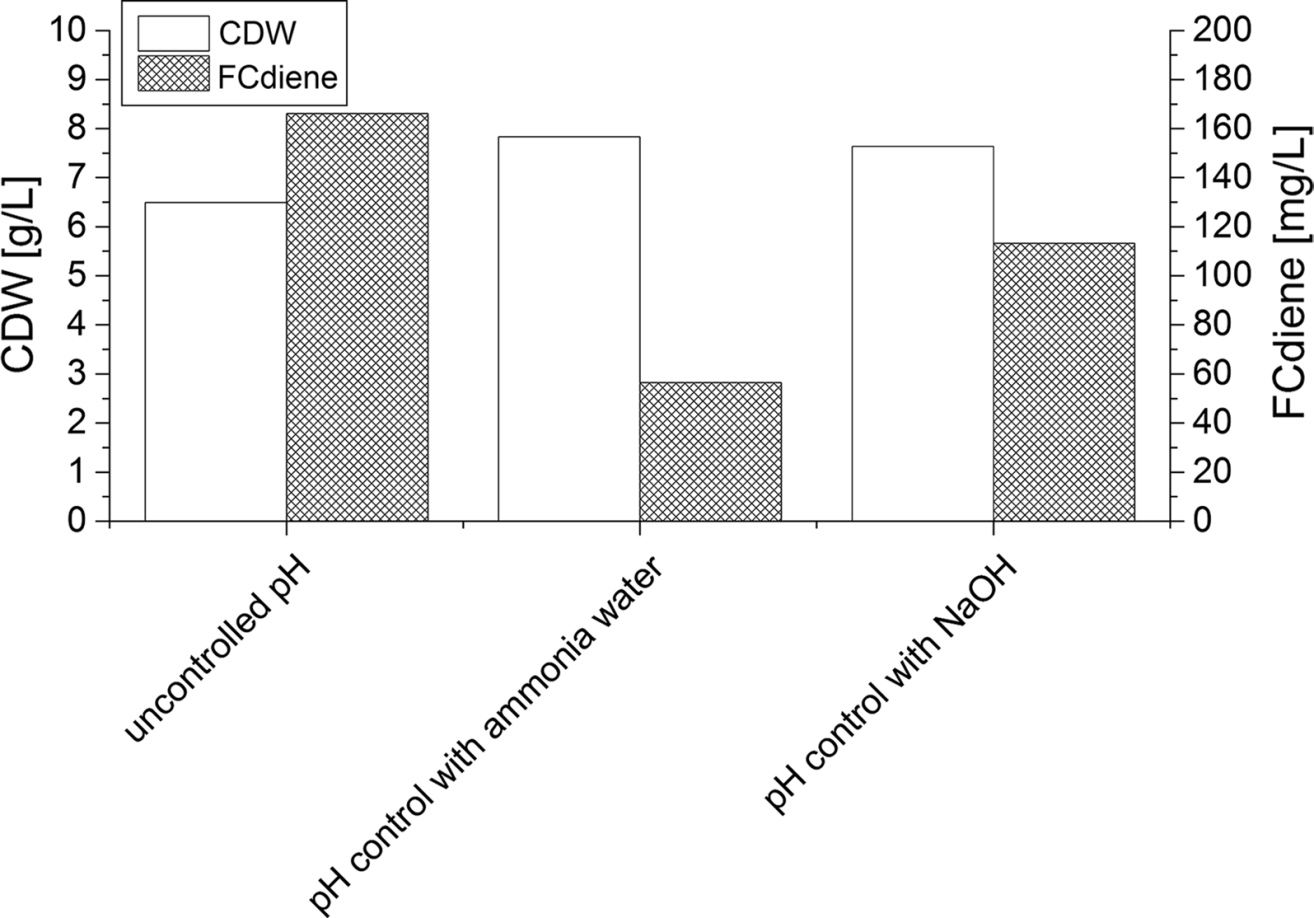
pH influence on fed-batch fermentations of *S. cerevisiae* in a KLF2000 fermenter (working volume 1.8 L), using glucose as a carbon source. Cell dry weight and FCdiene concentrations measured at the end of the fermentations are shown

As a next step, the pH influence on fed-batch fermentations was investigated to determine whether increasing biomass resulted in the observed FCdiene concentration increase when pH was kept constant. pH was regulated using ammonia solution (6.65 M) or NaOH (0.5 M). Ammonia solution was used to prevent nitrogen limitations. In addition, one fed-batch fermentation without pH regulation was performed (Fig. 6). The optimized feed and stirring profiles determined above were used.

A pH drop to 2.9 was measured which could be attributed to acid and CO_2_ production by the yeast. Biomass concentration of 6.7 g/L and FCdiene concentration of 166 mg/L were reached without pH regulation. The glucose concentration was variable around 0.03 g/L and a maximum value of up to 2.7 g/L ethanol was detected.

A pH between 4.3 and 4.8 could be regulated with ammonia solution. The biomass concentration increased up to 7.8 g/L, however, the maximal FCdiene concentration was ~ 47% lower compared to pH unregulated fermentation. The glucose concentration did not exceed 0.1 g/L, however ethanol concentration reached a maximum of 1.1 g/L. The ethanol concentration increased irrespective of glucose concentrations higher or lower than 0.1 g/L.

Using NaOH for pH regulation (pH 4.5), the biomass concentration reached 7.6 g/L, comparable to fermentations with ammonia solution pH regulation. Glucose concentration did not exceed 0.1 g/L but ethanol concentration rose until a maximum value of 1.5 g/L and 125 mg/L FCdiene was obtained.

### A bi-modal fermentation strategy

A bi-modal cultivation strategy was developed to further investigate results presented by Czarnotta et al. (2017). Czarnotta et al. (2017) resulted in higher product concentrations when ethanol was used as carbon source. It could be suspected that FCdiene is synthesized from acetyl-CoA which is synthesized from ethanol itself. Previous investigations in our group (Halka and Wichmann 2018) indicated higher FCdiene concentrations using glucose instead of ethanol as a carbon source in batch fermentations. Therefore, a new fermentation strategy combining batch and fed-batch mode was examined to determine if intracellular production of ethanol could be used to improve FCdiene yields after higher biomass concentrations from fed-batch fermentations were obtained.

A pH regulated fed-batch phase of 24 h was followed by a pH unregulated batch phase, in which ethanol production and consumption was permitted. The fed-batch was intended to generate increased biomass concentrations while the unregulated batch phase was intended to produce FCdiene, potentially supported by consumption of intracellular ethanol from Crabtree effect in this phase. An unregulated batch phase was used because higher FCdiene concentrations could be reached in previous unregulated batch fermentations (Halka and Wichmann 2018).

**Fig. 7:**
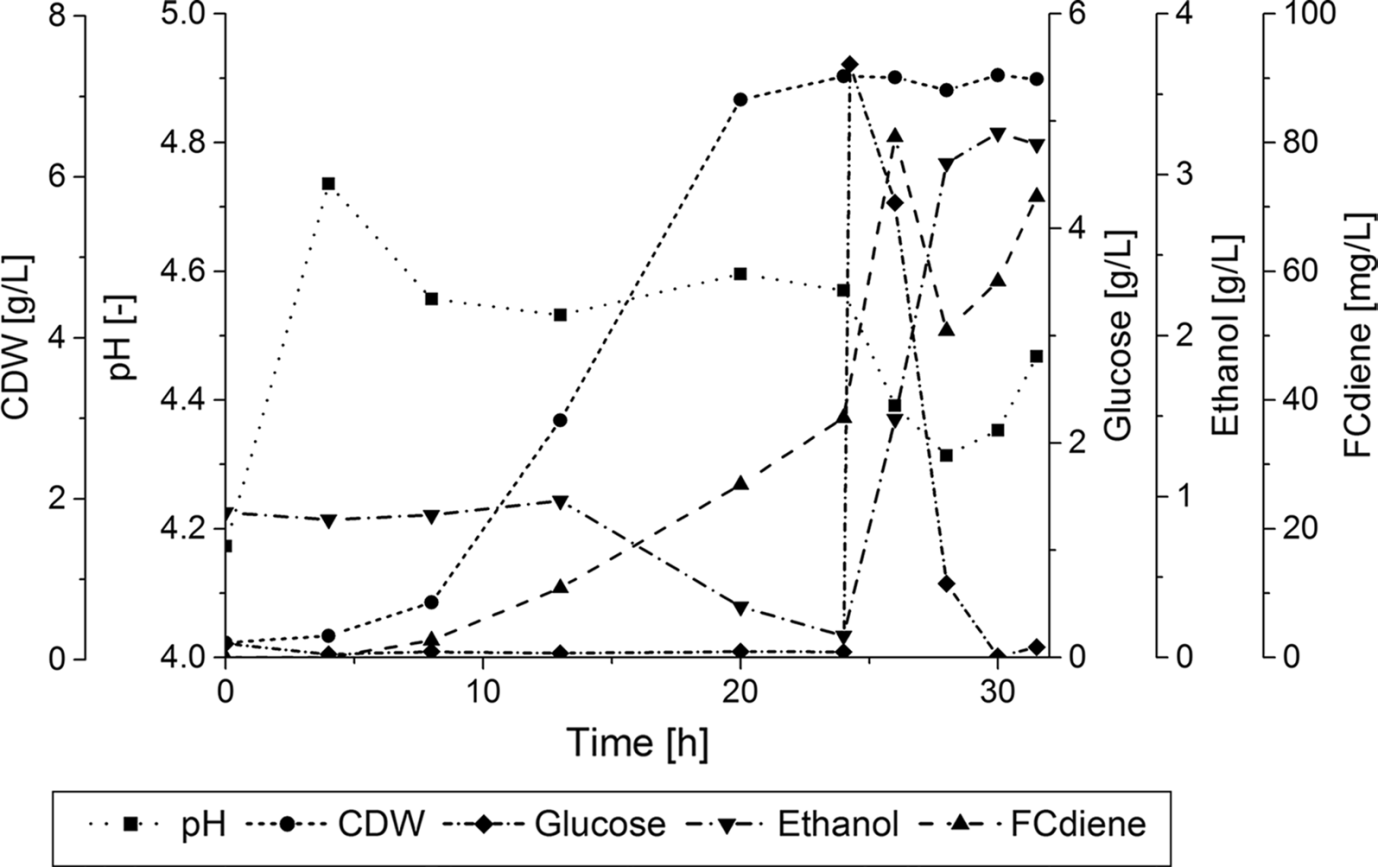
Bi-modal cultivation of *S. cerevisiae* growing in SD medium with glucose as sole carbon source. Cell dry weight concentrations, pH, glucose; ethanol and FCdiene concentrations of fed-batch fermentation (0–24 h) with combined glucose pulse in the KLF2000 fermenter (working volume 1.8 L) are shown. In the fed-batch phase, the pH was regulated using ammonia solution (6.65 M), in the batch phase the pH was unregulated

The cells were supplied with 5 g/L (0.03 M) glucose. The fermentation was performed in duplicate. One representative fermentation is depicted in Fig. 7, the other one is shown in Additional file 1: Figure S2. The biomass concentration was observed to reach 7 g/L, at 24 h additional glucose was added to the fermentation, and FCdiene concentrations (one outlier at 26 h) are indicated (Fig. 7). In this fermentation, the maximum FCdiene concentration reached only 72 mg/L an enhancing effect of use of intracellular ethanol in batch phase could not be measured.

## Discussion

In this study, two different fermentation strategies for increasing the fermentative production of heterologous FCdiene from *S. cerevisiae* were investigated. Fermentations using FeedBeads^®^ showed that glucose limitation increased the yields of FCdiene per substrate in fed-batch fermentations to 21.54 mg_FCdiene_/g_Glucose_. This yield represents 211.15% of the substrate specific FCdiene yield obtained in batch fermentation. Therefore, a fed-batch fermentation strategy with glucose and a bi-modal cultivation strategy were developed and performed.

A feed strategy used for 1.8 L scale fermentations which employed a variable µ was found to increase the biomass concentration up to 3X within the first 28 h of fermentation time, in comparison to batch fermentation (Table 1). The FCdiene concentration was observed to increase 2.8X within 28 h up to around 161 mg/L and increased up to 320 mg/L by 44 h, the highest reported FCdiene concentrations at this scale to date.

In the fed-batch fermentation with the staircase-shaped stirring profile, FCdiene concentrations over 400 mg/L were measured, however, this result could not be replicated. As shown in Table 1, there was no linear correlation between FCdiene production and growth rate or glucose consumption of the yeast. This indicates that the mechanisms responsible for FCdiene production are more complex than initially assumed. It may be that high FCdiene concentrations are the result of two specific factors which existed in parallel and reached an unknown optimum.

Because of the opposite inlets for glucose and sterile air supply, glucose is fed from the top and sterile air is supplied from the bottom of the reactor. There are possibly two gradients inside the reactor although stirring should prevent this. A glucose and a DOT gradient with highest glucose concentrations at the top of the fermenter and highest DOT at the bottom of the fermenter may occur. The glucose at the top is on the one hand converted to ethanol because of the Crabtree effect and on the other hand to FCdiene. Micro-aerobic conditions could be correlated with high FCdiene production (Mantzouridou et al. 2009). In this scenario, at the bottom of the reactor the FCdiene would be mainly produced based on ethanol metabolism because of higher oxygen concentrations. And now possibly both production ways reached a maximum at the same time and increased the FCdiene concentration up to 400 mg/L.

In this work, it could be shown that high product yields were achievable with manual and controlled continuous glucose feed. Substrate specific product yields up to 19 mg_FCdiene_/g_Glucose_ and cell specific product yields up to 0.36 mg_CDW_/g_Glucose_ were determined in this condition. The continuous glucose feed reached a substrate specific product yield up to 11 mg_FCdiene_/g_Glucose_ and a cell specific product yield of 0.23 mg_CDW_/g_Glc_.

250 mg/L FCdiene could be reproduced in several fermentation runs using fed-batch mode with manual or controlled glucose feed. This concentration is 12.5-fold higher than previously reported yields (Arens et al. 2014) and is as high as our results from batch shaking flasks (Halka and Wichmann 2018) which showed that a transfer to a scalable stirred tank reactor system is possible with similar results and shows more potential for the future. The combination of micro-aerobic fermentation conditions with the fed-batch mode indicates an additional increase in FCdiene concentrations. Batch fermentations showed an increase in FCdiene concentrations of 2.5–4.8X under micro-aerobic conditions (Halka and Wichmann 2018), therefore an increase in FCdiene concentrations of a similar degree is also to be expected for micro-aerobic fed-batch fermentations.

Investigating the influence of pH control on the FCdiene productivity in fed-batch fermentations showed a drop to 23% (NaOH) and 61% (ammonia water) in comparison to pH unregulated fermentations, supporting the earlier discussed negative influence of pH regulation on FCdiene production from glucose (Halka and Wichmann 2018). The observed larger decrease in FCdiene production when ammonia water was used as a pH correction agent may be due to the inherently higher nitrogen supply given by this buffer. This is in accordance with a study investigating the effect of nitrogen limitation on ergosterol production (Shang et al. 2006).

The production of FCdiene in *S. cerevisiae* did not correlate with growth of the yeast. It is possible that flux through the mevalonate pathway does not follow a linear correlation with culture growth, as the promoters used for the expression of transgenes in this host are constitutive, and should be continuously active (Arens 2015).

In accordance to previously described squalene production in *S. cerevisiae*, it could be possible that the FCdiene production is supported by ethanol consumption (Mantzouridou et al. 2009). When ethanol is metabolized by the cell, it is converted to acetyl-CoA which can enter the mevalonate pathway for generation of the terpene precursor isopentenyl pyrophosphate and dimethylallyl pyrophosphate (Czarnotta et al. 2017). The potential for enhanced FCdiene production led us to investigate whether a combination of fed-batch and batch modes could be used to boost FCdiene production rates by using ethanol from Crabtree effect. Additionally it was expected that increased growth in the fed-batch phase may increase the FCdiene production in the batch phase as well.

However, we observed no increased FCdiene production using this strategy, suggesting that accumulated biomass from batch phase does not necessarily behave as a directly pH unregulated batch culture. Here, the pH drop, which has been correlated to higher FCdiene production in batch cultures, was absent. In accordance with previously reported ergosterol production from *S. cerevisiae*, the FCdiene production here seemed to be inhibited by nitrogen concentrations of pH regulated fed-batch phase (Shang et al. 2006). For this reason a glucose fed-batch is actually the proposed strategy to generate reproducible high FCdiene product concentrations. As determined with H-NMR, purities of up to 80% were achieved from batch and fed-batch fermentations using pentane extraction.

The results of this study indicate that there is potential for more efficient FCdiene production by fed-batch fermentations using genetically modified *S. cerevisiae*, as yields presented here illustrate threefold improvement over former studies. The glucose feed strategy based on variable µ determined here appears to benefit terpene production from *S. cerevisiae.* It can replace a part of the chemical synthesis and may hold insights for other systems as well.

## Additional file

**Additional file 1.** Further methods and figures.

## Abbreviations

DMAPP: dimethylallyl pyrophosphate
DOT: dissolved oxygen tension
FCdiene: fusicocca-2,10(14)-diene
GC: gas-chromatograph
IPP: isopentenyl pyrophosphate
SD medium: synthetic dropout medium
SPE: solid phase extraction
tHMGR: truncated HMG-CoA reductase
