# Supplementary material for: Glucose limited feed strategy leads to increased production of fusicocca-2,10(14)-diene by Saccharomyces cerevisiae
Source: AMB Express. 2018 Aug 22;8:132. doi: 10.1186/s13568-018-0662-8 (PMC6104463; doi:10.1186/s13568-018-0662-8)
Supplement: Supplementary file 1 — Additional file 1. Further methods and figures. [file 13568_2018_662_MOESM1_ESM.pdf]

Additional file 1

Further methods and figures

Glucose limitation feed strategy leads to increased production of fusicocca-2.10(14)-diene by *Saccharomyces cerevisiae*

AMB Express

Lisa Marie Halka, Christian Nowacki, Alica Kleinschmidt, Kevin Koenen and Rolf Wichmann[a]

[a] TU Dortmund University, Department of Biochemical and Chemical Engineering, Laboratory of Biochemical Engineering, Emil-Figge-Straße 66, 44227 Dortmund, Germany. rolf.wichmann@tu-dortmund.de

Materials and methods

Optical density and cell dry weight

The optical density (OD) of fermentation samples was measured using a spectrophotometer Libra S11 (Biochrom Ltd, Cambridge, United Kingdom) at a wavelength of λ = 600 nm. The cell dry weight (CDW) of *S. cerevisiae* CEN.PK2-1c [pRS313-*upc2.1*, pRS315-*thmgr*, pVV214-*abfs*] (Arens et al. 2014) was calculated with the following correlation:

$$CDW\ \left[\frac{g_{CDW}}{L}\right] = 0.2539\ \cdot OD_{600}$$

pH measurement

For shake flask experiments, the pH of each fermentation sample was measured after cell separation at 16,200 x g for 10 min at room temperature. For fermentation processes, in a 3.1 L glass KLF 2000 fermenter, the pH was measured and regulated in-line with a pH controller (Bioengineering AG, Wald, Switzerland), a SteamLine pH electrode (SI Analytics GmbH, Mainz, Germany) and a peristaltic pump for bases.

Glucose and ethanol quantification

Glucose and ethanol concentrations were measured using a High Pressure Liquid Chromatography (HPLC) Smartline system consisting of a pump 1000, a Oven Jet Stream 2 Plus and a RI-detector 2300. The pre column (30 x 8 mm, 10 µm) and the main column (300 x 8 mm, 10 µm) were both Vertex Plus Columns Eurokat® H (Dr.-Ing. Herbert Knauer GmbH, Berlin, Germany). A method with constant temperature of 65 °C was used. The flow rate of the mobile phase (0.5 mM sulfuric acid) was set to 0.6 mL/min with a run time of 32 min.

Figures

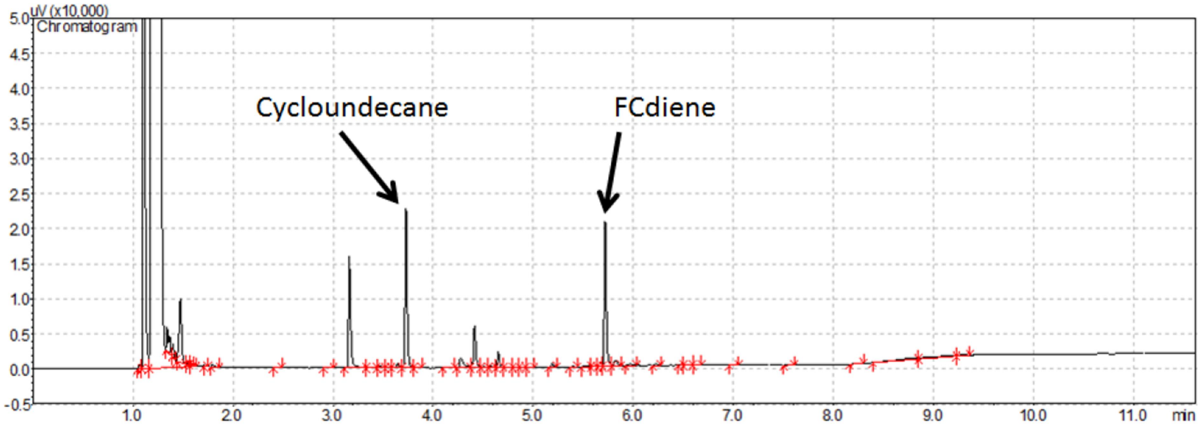

Figure S1 Chromatogram of a fermentation sample of *S. cerevisiae* growing in SD medium with glucose as a sole carbon source after new product recovery with SPE. The intensity as a function of retention time is shown. Cycloundecane used as an internal standard and the product FCdiene are marked.

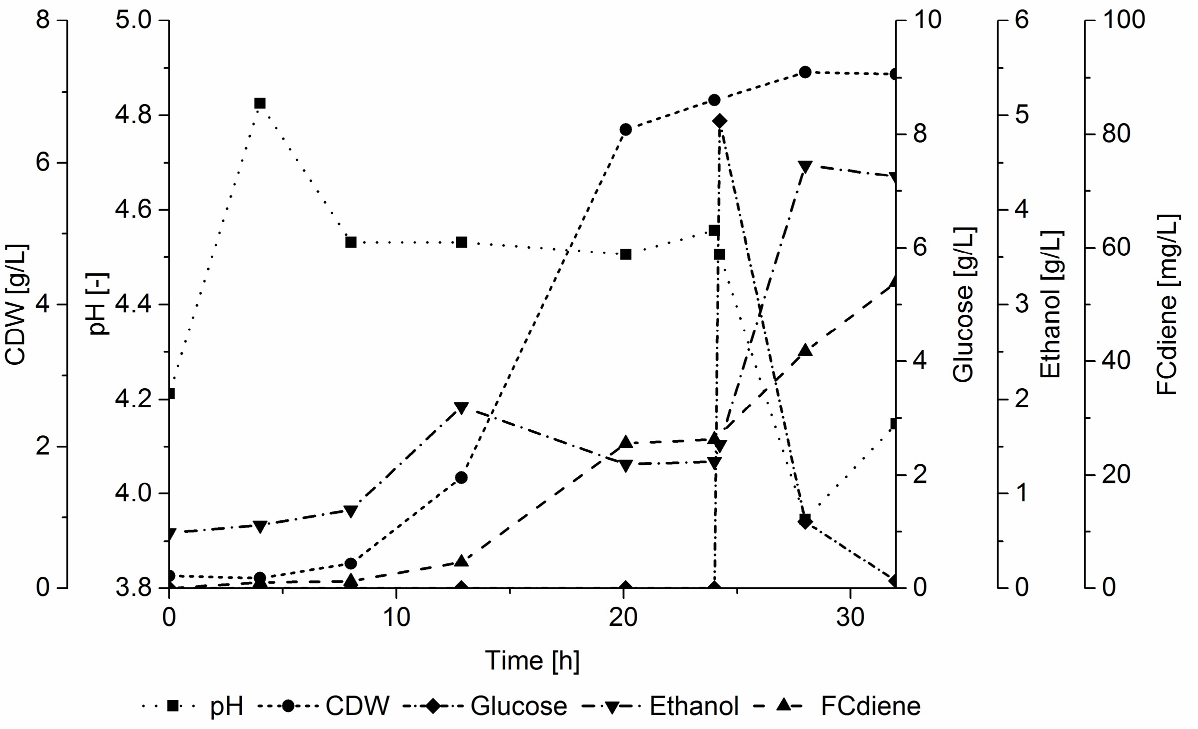

Figure S2 Second bi-modal cultivation of *S. cerevisiae* growing in SD medium with glucose as sole carbon source. Cell dry weight concentrations, pH, glucose; ethanol and FCdiene concentrations of fed-batch fermentation (0-24 hours) with combined glucose pulse in the KLF2000 fermenter (working volume 1.8 L) are shown. In the fed-batch phase, the pH was regulated using ammonia solution (6.65 M), in the batch phase the pH was unregulated.
